# Medical Society of the County of Chemung

**Published:** 1911-02

**Authors:** Charles Haase


### Medical Society of the County of Chemung.

REPORTED BY CHARLES HAASE, M.D., Secretary.

At the annual meeting of the Medical Society of the County of Chemung held at Elmira, December 20, 1910, the following resolution was adopted:

Whereas, The Medical Society of the State of New York in 1909 lost $6,053.09 in publishing the Tri-State Medical Directory and it loses a like amount each year; and

Whereas, If the state society discontinued the publication of the directory it would save this annual expense, and as a consequence we could reduce our state dues. The state society now receives three of the four dollars we each pay for annual dues, therefore,

*Resolved*, That the Medical Society of the County of Chemung do all in its power to have the state society discontinue the publication of the directory and that it instruct its state delegate to work for this measure.

Section three of the A. M. A. medical directory contains ten states including New York and sells for two dollars.

---

Memorial Address on Dr. Robert Koch.—John A. Wyeth, New York, gave a synopsis of the life of Dr. Robert Koch and his work in bacteriology. Koch was both a worker and a thinker, and should be reckoned among the heroes of the world.—*Medical Record*, January 21, 1911.

